# Efficient Extraction and Structural Characterization of Hemicellulose from Sugarcane Bagasse Pith

**DOI:** 10.3390/polym12030608

**Published:** 2020-03-06

**Authors:** Yitong Xie, Xin Guo, Zhiyu Ma, Jingwei Gong, Haisong Wang, Yanna Lv

**Affiliations:** 1 School of Light Industry and Chemical Engineering, Dalian Polytechnic University, Dalian 116034, China; yitong_x@163.com (Y.X); gx9255@163.com (X.G.); mazhiyu_dlpu@163.com (Z.M.); hbdyygjw@163.com (J.G.); 2 State Key Laboratory of Pulp and Paper Engineering, South China University of Technology, Guangzhou 510640, China

**Keywords:** sugarcane bagasse pith, hemicellulose, ultrasound-assisted alkaline extraction, response surface methodology, structural properties

## Abstract

The aim of this study was to investigate the ultrasound-assisted alkaline extraction process and structural properties of hemicellulose from sugarcane bagasse pith. Response surface model (RSM) was established in order to optimize the extraction conditions for the highest hemicellulose yield based on the single-factor experiments. A maximum total hemicellulose yield of 23.05% was obtained under the optimal conditions of ultrasonic treatment time of 28 min, KOH mass concentration of 3.7%, and extraction temperature of 53 °C, and it evidently increased 3.24% compared without ultrasound-assisted extraction. The obtained hemicellulose was analyzed by Fourier transform infrared (FT-IR) spectroscopy. The monosaccharide composition and average molecular weight of hemicellulose were characterized by using ion chromatography (IC) and gel permeation chromatography (GPC). The results indicated that xylose was dominant component in water-soluble hemicellulose (WH, 69.05%) and alkali-soluble hemicellulose (AH, 85.83%), respectively. Furthermore, the monosaccharides (otherwise xylose) and uronic acids contents of WH were higher than that of AH. Weight average molecular weight of WH was 29923 g/mol, lower than that of AH (74,872 g/mol). These results indicate that ultrasonic-assisted alkaline extraction is an efficient approach for the separation of hemicellulose from sugarcane bagasse pith.

## 1. Introduction

Sugarcane bagasse is one of the most important available by-products of sugarcane extraction, consisting of fiber bundles and other structural elements like vessels and parenchyma, which can be summarized under the technical term pith [1,2,3]. As 30% to 40% pith of bagasse causes a series of problems, including low pulp yield, high chemical consumption, and pith-related pulp quality problems in pulp and papermaking, it should be removed as far as possible [4,5,6]. In China, the annual output of sugarcane is 100 million tons [6,7], of which nearly 23% will be converted into bagasse. Therefore, approximately 23 million tons of sugarcane bagasse and 8 million tons of bagasse pith are produced. The impressively abundant bagasse pith is mainly utilized as fuel or a feedstuff, but it can be used for more value-added products.

Bagasse pith (BP) is formed by three main components: cellulose, hemicellulose, and lignin [8]. Hemicellulose is an amorphous chain of polysaccharides, which can be converted into fermentable sugars after pretreatment and hydrolysis and produce bioethanol and biochemical [9,10]. In addition, hemicelluloses can be regarded as film forming materials, hydrogel, binders, and emulsifiers in food, packaging, and biomedical industries [11,12]. Therefore, extracting hemicelluloses from BP and retaining their characteristic structure as much as possible is very important for efficient utility of substance. However, lignin and hemicelluloses are linked by covalent bonds, forming the lignin carbohydrate complex (LCC) [9]. Beyond that, hemicelluloses combine closely with cellulose by hydrogen bonds. These bonds restrict hemicelluloses isolate from the plant cell walls [13]. Therefore, it is promising to develop an efficient extraction method to increase the hemicellulose extraction yield and purity.

A number of extraction methods, such as dilute acid extraction, alkali extraction, hot water extraction, and organic solvent extraction have been applied to extracting hemicelluloses [14]. Among these, alkali extraction has been commonly used due to its cost-effective and convenient operation [15]. Recently, ultrasound-assisted extraction has received great interest in the isolation of polysaccharides from plants because of its high efficiency, lower energy input and environment-friendly [16,17]. The mechanism of the ultrasonic treatment is that ultrasonic wave produces a special cavitation effect in aqueous medium, which can destroy cell wall structure [15,18,19]. In previous studies, the application of ultrasound showed an effective effect on the alkaline extraction of hemicelluloses, the obtained hemicellulose with high yields [20] complete structure, high degree of polymerization, and high molecular weight [21,22]. Besides, hemicellulose extracted by KOH has high purity compared to that by NaOH [23,24]. Although, there are many literatures reporting the extraction of hemicellulose from bamboo, corn cob, and bagasse combining alkali solution and ultrasound treatments, few researches have focused on the extraction of hemicellulose from BP by using a combination of alkali solution and ultrasound-assisted extraction.

Within this context, the main aim of this study was to optimize ultrasonic time, KOH mass concentration, and extraction temperature on ultrasound-assisted alkaline extraction by response surface methodology (RSM) with the intention to maximize the extraction yield of hemicellulose. Meanwhile, the structural properties of water-soluble hemicellulose (WH) and alkali-soluble hemicellulose (AH) from BP were initially characterized by Fourier transform infrared (FT-IR), ion chromatography (IC), and gel permeation chromatography (GPC). It may provide fundamental information for further utilization of BP hemicellulose.

## 2. Materials and Methods

### 2.1. Raw Materials 

BP was obtained from Guangxi Laibin East Sugar Paper Co., Ltd. (Laibin, China). The fraction of BP that could pass a 40-mesh screen was used in all experiments. The chemical compositions of BP were determined according to the the following Tappi Test Methods. The results and corresponding standards were shown in Table 1. The holocellulose was prepared by treating benzene-ethanol extractive BP with NaClO_2_ solution and glacial acetic acid at 75 °C for 1 h, which repeated for three times.

### 2.2. Ultrasound-Assisted Extraction and Isolation of Bagasse Pith

**Figure 1:**
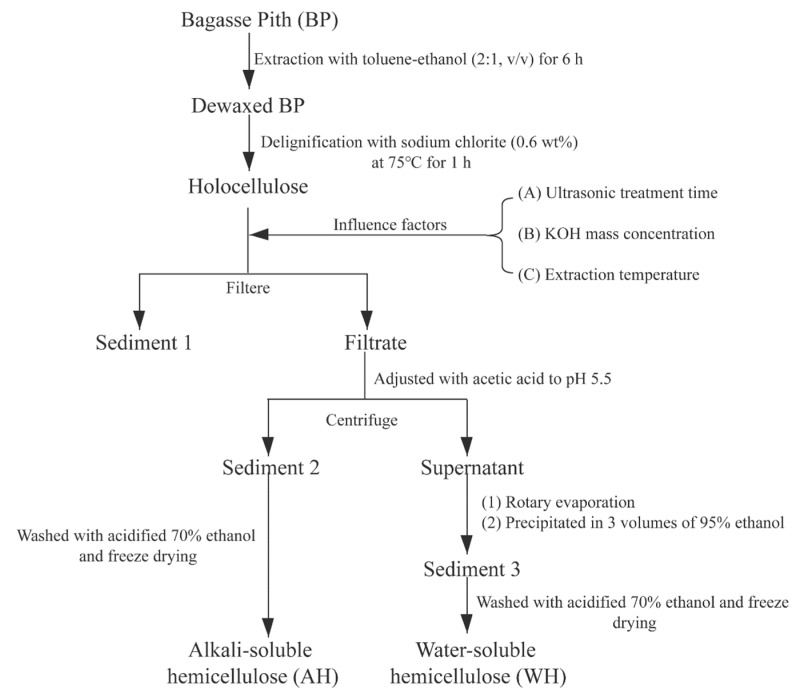
Scheme for extraction of hemicelluloses from BP.

The dried BP raw material was first extracted with toluene-ethanol (2:1, v/v) in a Soxhlet apparatus for 6 h, and the obtained material was allowed to dry at 105 °C for 12 h. Then, the dewaxed sample was delignified with sodium chlorite (0.6 wt %) at 75 °C for 1 h and repeated three times. After the treatment, the residue (holocellulose) was filtered through glass fliters (1G2) and washed with deionized water to neutral. Subsequently, the holocellulose was extracted with a certain mass ratio KOH solution under different ultrasonic time. Ultrasonic processing was executed in the ultrasonic cleaner (SK2510 HP, Shanghai Kudos ultrasonic instruments Co., Ltd., Shanghai, China), equipped with a fixed ultrasound power (250 W) and adjustable frequency (40%–100%, 53 kHz). The mixture was then treated with the KOH solution under continuous stir at constant temperature for 2 h. The filtrate was adjusted to pH 5.5 with acetic acid. AH was obtained by centrifuging, washing with 70% ethanol, and freeze drying. WH was from the supernatant after centrifuging; it was also collected by precipitating the concentrated filtrates with 3 volumes of 95% ethanol. The scheme for fractionation of hemicelluloses from BP is illustrated in Figure 1. At the same time, compare the hemicellulose yield treated with 3.7% KOH solution at 53 °C for 30 min with those treated without ultrasound. The resulting WH and AH require freeze-dried. The yield of AH and WH is calculated as follows,

(1) $$Y(\%)=\frac{m}{\frac{m_{0}^{}}{1-X}}\times 100\%$$

where, *Y* is AH (or WH) yield (%), *m* is the mass of freeze-dried AH or WH (g), *m*_0_ is the mass of absolute dry BP after Benzol extraction (g), *X* is the content of Benzol extractive (%). The total yield of BP hemicellulose is the sum of AH yield and WH yield.

### 2.3. Response Surface Methodology Design

On the basis of single-factor experiments, optimization of ultrasound-assisted alkali treatment of the BP was implemented by using Box–Behnken Design, generated by Design-expert 8.0.6 software (Stat-Ease Inc., Minneapolis, MN, USA). The effects of three independent variables of the response were investigated. The variables used were ultrasonic treatment time (A: 20–30 min), KOH mass concentration (B: 2–4 wt %), and extraction temperature (C: 40–60 °C) (Table A1), when the total hemicellulose yield was considered as response. A total of 17 experiments were conducted, including 12 factorial experiments and 5 central point repeats. The parameters were optimized by Design-expert software, obtaining the maximum hemicellulose yield. Three-dimensional and two-dimensional response surface plots were carried out for analyzing the mutual influence among ultrasonic treatment time, KOH mass concentration, and extraction temperature.

### 2.4. Infrared Analysis

FT-IR were measured on Spectrum 10 spectrometer (PerkinElmer Inc., Waltham, MA, USA), and frequency range of 500~4000 cm^−1^. The hemicellulose samples with KBr were at a ratio of 1:100 and pressed the mixture to flake.

### 2.5. Sugar Analysis

The sugar and uronic acids content of hemicellulose was determined by using an Ion Chromatography (IC) unit equipped with CarboPacTM PA1 column (Dionex-300, Dionex Corporation, Sunnyvale, CA, USA) and a pulsed amperometeic detector (PAD) according to the procedure presented by Cui and Saeed [25,26]. Hemicellulose samples were hydrolyzed with 4 v/v% H_2_SO_4_ solution at 121 °C in Autoclave of Uniclave Series (GI80DS, Zealway Instrument INC, Xiamen, China) for 120 min to convert to sugar and uronic acids. The PAD setting for the determination was E1 = 0.1 V, E2 = 0.6 V, and E3 = −0.8 V. The eluent was a mixture of 8 g L^−1^ NaOH and 20 g L^−1^ CH_3_COONa, and the flow rate was 1 mL/min. Hemicellulose samples were diluted and filtered by 0.45 μm filter head.

### 2.6. Content of Residual Lignin

The total residual lignin in obtained hemicellulose was determined as the sum of acid soluble and klason lignin. The acid soluble lignin and klason lignin content were determined by TAPPI method T250 and TAPPI method T222, respectively.

### 2.7. Molecular Weight Determination

The molecular weight of the AH and WH was estimated using the procedure described by Xu and Li [27,28] by gel permeation chromatography (GPC) equipped with waters 2414 Refractive Index Detector (RID). The TSK-GEL columns (Tosoh Co., Ltd, Tokyo, Japan) in series were G-5000 PW_XL_ (7.8 mm × 300 mm) and G-3000PW_XL_ (7.8 mm × 300 mm). The column was eluted with 0.02 mol/L KH_2_PO_4_ aqueous solution at a flow rate of 0.6 mL/min and calibrated with the Dextran standards (average molecular weights of 5200; 11,600; 23,800; 148,000; 273,000; 410,000; and 668,000 g/mol). The temperature of the columns was maintained at 35 ± 0.1 °C. In each run, the volume of injection was 20 µL.

## 3. Results and Discussion

### 3.1. Response Surface Analysis

The coded values of three individual variables and total BP hemicellulose yield under different conditions were shown in Table 2. A second-order polynomial equation in terms of Coded Factors for total hemicellulose yield can be represented by Equation (1):

Equation (1) in Terms of Coded Factors:

Y = 21.94 − 0.56 A + 2.17 B + 0.92 C + 0.09 AB − 0.96 AC + 0.47 BC − 2.99 A^2^ − 1.76 B^2^ − 1.05 C^2^ (2)

where Y is the predicted total yield of hemicellulose; A, B, and C are the coded values for ultrasonic treatment time, KOH mass concentration, and extraction temperature, respectively, and so as follows.

The experimental conditions and results of 17 runs are shown in Table A2. The results of analysis of variance (ANOVA) are presented in Table A2. *p*-values and F-values are used to verify the significance between coefficients. Specifically, Prob > F values less than 0.01 or 0.05 indicate that coefficients of the model are significant, and the values more than 0.05 indicate that coefficients of the model are not significant [29,30]. Therefore, F Value of 63.77 and Prob > F of less than 0.01 show that the model is significant, and there is only a 0.01% chance of such a large “Model F-value” due to noise in the model. Additionally, the linear coefficients (A, B, C), the quadratic term coefficients (A^2^, B^2^, C^2^), and the interaction of AC between ultrasonic treatment time and extraction temperature were all significant at the lever of *p* < 0.05 or *p* < 0.01, and the other coefficients are not significant. Therefore, the coefficients of A, B, C, A^2^, B^2^, C^2^, and AC were crucial factors during the BP extraction process. Above of all, it is confirmed that the experimental model is successful, and the regression equation could be used to predict the effects of ultrasonic treatment time, KOH mass fraction, and extraction temperature on BP hemicellulose yield [14,31]. The final equation in terms of actual factors can be described as follows:

Equation (2) in Terms of Actual Factors:

Y = −62.85 + 2.19 A + 10.08 B + 1.28 C + 0.009 AB − 0.0096 AC + 0.047 BC − 0.0299 A^2^ − 1.76 B^2^ − 0.0105 C^2^ (3)

**Figure 2:**
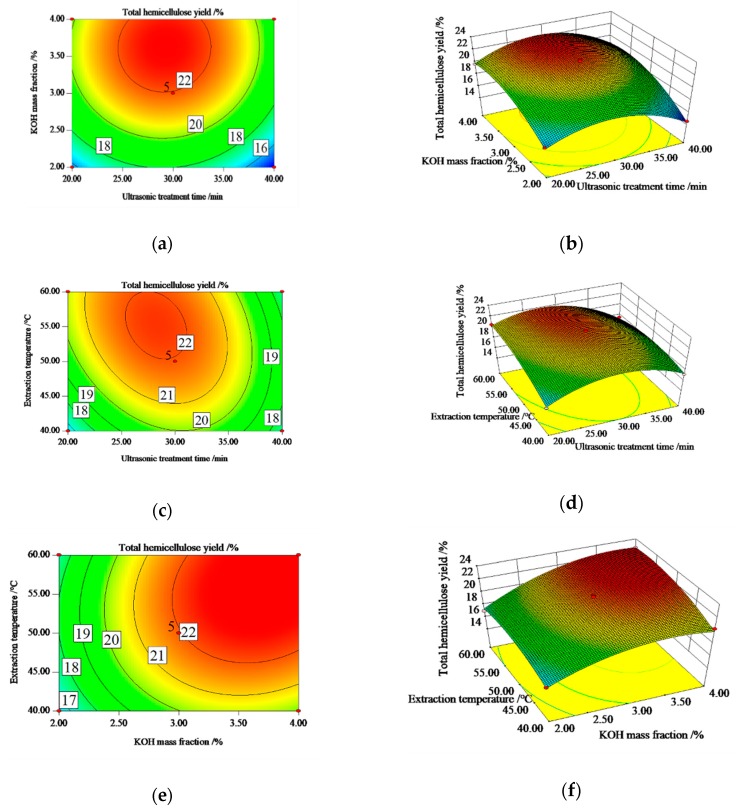
Response surface and contour plots showing effects extraction temperature, ultrasonic treatment time and KOH mass fraction on total hemicellulose of BP. (**a**) and (**b**) ultrasonic treatment time vs. KOH mass fraction (extraction temperature = 50 °C). (**c**) and (**d**) extraction temperature vs. ultrasonic treatment time (KOH mass fraction = 3%). (**e**) and (**f**) KOH mass fraction vs. ultrasonic treatment time (temperature = 30 min).

As illustrated in Figure 2, two-dimensional and three-dimensional graphs are the curve representations for regression models, which were helpful to predict the interaction of each two variables on total hemicellulose yield. The circle center of contour map is the condition where the extremum is. The closer the contour map curve is to a circle, the lower correlation between the two factors is, and the closer it is to the ellipse, the higher the correlation between the two factors is [28]. Response surface analysis of three-dimensional graphs is primarily based on the density and shape of contour plots. In sharp contrast, steep curve areas showed much more significant effects on response value. 

Figure 2a,b shows the effects between KOH mass concentration and ultrasonic treatment time on the total hemicellulose yield under the conditions of extraction temperature of 50 °C. The total yield increased at first and then decreased with the values of two variables increasing. From Figure 2a, the density of contour plot with the change of KOH mass concentration was larger than that of ultrasonic treatment time, which illustrated that effects of KOH mass concentrations on the hemicellulose yield were more significant than that of ultrasonic treatment time, and the *p*-value of regression analysis also reflected this. From Figure 2b, the contour plot was more like a circle, indicating a low correlation between the factors of KOH mass concentration and ultrasonic treatment time, and the *p*-value of AB in regression analysis also reflected that.

Figure 2c,d indicates the impacts of ultrasonic treatment time and extraction temperature on the total hemicellulose yield under the conditions of KOH mass concentration of 3%. The total yield increased at first and then decreased with the values of two variables increasing. From Figure 2c, the density of contour plot with the change of extraction temperature was larger than that of ultrasonic treatment time, which indicated that the effects of extraction temperature on the hemicellulose yield were more significant than that of ultrasonic treatment time, and the *p*-value of regression analysis also reflected this. The contour plot was more like an ellipse, suggesting a high correlation between the factors of extraction temperature and ultrasonic treatment time, and the *p*-value of AC in regression analysis also reflected that. 

Figure 2e,f reveals the interaction of KOH mass concentration and extraction temperature on the total hemicellulose yield under the conditions of ultrasonic treatment time of 30 min. From Figure 2e, the density of contour plot with the change of KOH mass concentration was larger than that of extraction temperature, which indicated that effects of KOH mass concentration on the hemicellulose yield were more significant than that of extraction temperature, and *p*-value of regression analysis also reflected this. The contour plot was more like a circle, indicating a low relation between the factors of KOH mass concentration and extraction temperature, and *p*-value of BC in regression analysis also reflected that.

The BP hemicellulose yield was optimized by Design-expert 8.0.6 software, and the optimum levels of the variables were ultrasonic treatment time of 28 min, KOH mass concentration of 3.7%, and extraction temperature of 53 °C, obtaining the predicted yield of 23.07%. According to the optimum conditions, the obtained hemicellulose yield was 23.05%, equivalent to the predicted value of the model, which indicates this model can be used to predict BP hemicellulose yield.

### 3.2. FT-IR Spectra 

**Figure 3:**
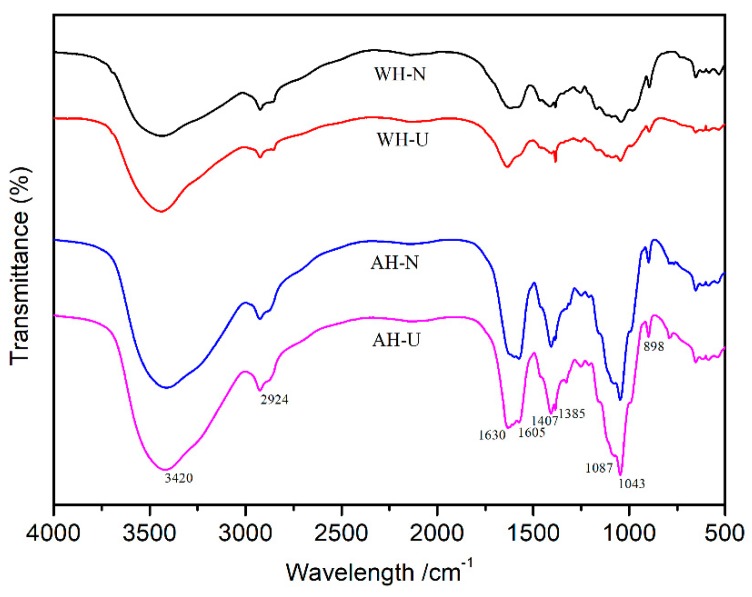
FT-IR spectra of WH-N, WH-U, AH-N, and AH-U.

The FT-IR spectra of AH and WH were illustrated in Figure 3. The following analyses were based on previous reports [20,28,32,33]. It can be seen that the typical peaks of hemicelluloses were 3420, 1630, 1407, 1043, and 898 cm^−1^ [34,35]. The peaks of 3420 and 2924 cm^−1^ were ascribed to the O–H and C–H stretching of hemicellulose, respectively, while the absorption peak at 1407 cm^−1^ was C–H stretching, and the absorption peak at 1043 cm^−1^ corresponded to the C–O, C–C stretching or C–OH bending of typical xylose pyran ring. The characteristic band at 898 cm^−1^ assigned to the symmetric stretching vibration of C–O–C, indicating that xylan structure was linked by β-1,4 glycosidic bonds [36,37]. A characteristic band at 1243 cm^−1^ appeared corresponding to O–H or C–O–C bending, which confirmed the existence of xylan. Otherwise, there were no significant differences on the structure of AH and WH from Fourier-transform infrared spectra after ultrasound assisted treatment, demonstrating that ultrasonic treatment was a physical process, which keeps the basic chemistry bonds intact after ultrasound treatment.

### 3.3. The Effect of Ultrasound-Assisted Extraction on Hemicellulose Yield and Sugar Analysis

The hemicellulose yields and monosaccharide compositions of WH and AH are implied in Table 3. The hemicellulose extracted by alkaline extraction by means of ultrasound-assistant has a much higher yield, which is 23.05%, compared to that of without ultrasound-assisted extraction that is 19.81%. These results were higher than those reported by Liu [38]: under conditions of NaOH charge 10%, extraction temperature of 120 °C, extraction time of 60 min, the hemicellulose yield of bagasse pith was 15.37%. In addition, several researchers have investigated the positive effect of ultrasonic treatment on the hemicellulose extraction yield from different agricultural biomass. A total of 41.4% of the wheat straw hemicelluloses were obtained (absolute yield increase by 1.8 using ultrasound) [39], and 16.5% total buckwheat hulls hemicelluloses were isolated (absolute yield increase by 5.3% using ultrasound) [20]. Moreover, the yield of AH and WH treated with ultrasound-assisted extraction is higher than that of without ultrasound-assisted extraction. It demonstrates that ultrasonic treatment is suitable for solubilizing hemicellulose. The total yield of hemicellulose increased from 19.81% to 23.05% when the ultrasonic treatment time was increased to 28 min. Making BP cell walls swell and hydrate may result from the cavition of ultrasound [27]. This also makes it easier for the KOH solution to reach the BP cell wall. Beyond that, ultrasound can loosen and destroy the chemical bonds among hemicellulose, cellulose, lignin and other substances, making hemicellulose filled with alkali solution, then accelerating the dissolution of hemicellulose, which could also be attributed to the growth of hemicellulose yield. During the ultrasound-assisted process, there is a section of hemicellulose degradation, which may be caused by radical reaction induced by ultrasonic treatment, resulting in the fracture of glycosidic bonds among the sugars [28,40,41]. In the process of ultrasonic assistance, the extraction yield of hemicellulose has increased a lot with low energy consumption of ultrasound. In our work, a fabricated method for industrial extraction and separation of hemicellulose from agricultural and forest wastes is developed, that is, ultrasound-assisted alkaline, and it has a promising application [42]. 

Table 3 also shows that xylose was the dominant component sugar in WH and AH, whether or not they were ultrasound assisted, which was followed by arabinose and glucose. Small amounts of galactose and glucuronic acid and minor amounts of galacturonic acid were observed as well. Nevertheless, some differences of chemical composition between the two hemicellulosic components were observed. The content of xylose was up to 85.83% of the total fractions in the AH obtained by sonication. In contrast to AH, the content of xylose in WH was only 69.05% of the total fractions, whereas other hemicellulosic mosaccharides were also significant in WH. The ratio of arabinose to xylose (Ara/Xyl) reflected the degree of branching of hemicellulose, and the higher ratio indicated the higher degree of branching [24]. The Ara/Xyl ratios of WH were higher than those of AH, which suggested the AH was more homogeneous than WH.

In addition, the xylose content of the hemicellulose obtained by ultrasonic treatment was slightly lower than that of without ultrasound. This was the opposite as the variation of arabinose and galactose. The results might be attribute to the fact that the various cell walls position of hemicellulose components and the different accessibility of ultrasound-assisted alkaline extraction. The effect of ultrasound on BP cell wall can improve cell accessibility, contributing to an increase of sugar content [28]. Besides, the Ara/Xyl ratio of WH by ultrasonic treatment was higher than that of without ultrasound. This indicated that ultrasonic treatment had no apparent effect on original molecular structure of hemicellulose from BP. Since more hydrophilic sidechains were kept from WH, they were more active in chemical reaction, which was good for hemicellulose modification and for producing functional polymers.

### 3.4. The Effect of Ultrasound-Assisted Extraction on Content of Residual Lignin

The lignin of dewaxed BP was removed with sodium chlorite on the first step during the BP extraction process. Nevertheless, there was still residual lignin in obtained hemicellulose. It is well known that lignin in the cell wall of gramineous plants links arabinose or xylosyl via ester and aryl-ether bonds [43,44,45]. From Table 4, the content of residual lignin in AH was lower than that of in WH, which might be attribute to the fact that lignin mainly links with arabinose in BP cells, and the content of arabinose in AH is lower than that of in WH. The application of ultrasound resulted in a decrease in the content of residual lignin in hemicellulose, which can be explained by mechanical disruption of the ultrasound on bagasse pith cell walls resulting in the increased alkali solution accessibility and number of broken covalent bonds between hemicellulose and lignin [46]. Similar results have been reported by Sun and Tomkinson [39] during the extraction of hemicellulose from ultrasound-treated wheat straw. Therefore, ultrasound-assisted alkaline extraction optimized by response surface method could further reduce the content of residual lignin in obtained hemicellulos and improve the purity of hemicellulose.

### 3.5. The Effect of Ultrasound-Assisted Extraction on Molecular Weight

The average molecular weight of AH and WH was determined to explore if the ultrasound treatment could lead to a degradation of hemicellulose [27]. Weight-average (*M*_w_) and number-average (*M_n_*) molecular weight, as well as the polydispersity (*M*_w_/*M*_n_) of AH and WH were presented in Table 5. Hemicellulose is a heterogeneous glycan with polydispersity. The higher of polydispersity coefficient (*M*_w_/*M*_n_) it is, the wider of the hemicellulose molecular weight distribution range it is. The number average molecular weight and the weight average molecular weight of AH and WH decreased after ultrasonic treatment, the same variation trend was discussed by Xu et al. [27], which may be due to the fact that ultrasound was easy to break the glycosidic bonds, resulting in the degradation of hemicelluloses [39,40,41]. On the other hand, ultrasonic treatment can dissolve most of the lignin, and the residual lignin content of hemicellulose was tiny, subsequently, reducing the relative molecular weight of hemicellulose [32].

## 4. Conclusions

The ultrasound-assisted treatment was an effective method for the yield and efficiency of hemicellulose extraction from sugarcane bagasse pith. Extraction conditions optimized by RSM were ultrasonic treatment time of 28 min, KOH mass concentration of 3.7%, and extraction temperature of 53 °C. Under these conditions, total hemicellulose yield was 23.05%, and it evidently increased 3.24% compared with that without ultrasound-assisted extraction. In addition, the obtained hemicellulose showed a lower content of residual lignin, a slightly lower content of xylose, and lower weight average molecular weight. On the other hand, hemicellulose structure was integrated after ultrasound-assisted treatment from FT-IR spectra. Sugar analysis showed that AH and WH were mainly composed of both xylan and arabinose, and AH was more homogeneous than WH. Weight-average molecular weight of AH is higher than that of WH. Therefore, the ultrasound-assisted alkali extraction was suitable for preparation of hemicellulose from industrial waste bagasse pith.

## Figures and Tables

**Table 1:** Chemical composition of bagasse pith (BP).

Content (%)	BP	Standard
Ash	4.50	T211 om12
1%NaOH extractive	41.57	T212 om88
Benzol extractive	2.74	T204 om88
Holocellulose	69.49	GB/T 2677.10 (1995)
Pentosan	21.14	T223 om84
Klason lignin	20.28	T222 om88
Acid soluble lignin	1.95	T250

**Table 2:** Analysis of variance and significance testing.

	Sum of Squares	Df (Degree of Freedom)	Mean Square	F Value	*p*-Value Prob > F
Model	111.93	9	12.44	63.77	<0.0001
A-A	2.51	1	2.51	12.87	0.0089
B-B	37.71	1	37.71	193.40	<0.0001
C-C	6.75	1	6.75	34.63	0.0006
AB	0.032	1	0.032	0.17	0.6957
AC	3.69	1	3.69	18.9	0.0034
BC	0.89	1	0.89	4.58	0.0696
A^2^	37.61	1	37.61	192.87	<0.0001
B^2^	12.99	1	12.99	66.60	<0.0001
C^2^	4.61	1	4.61	23.64	0.0018
Residual	1.37	7	0.20		
Lack of Fit	0.64	3	0.21	1.16	0.4272
Pure Error	0.73	4	0.18		
Cor Total	113.29	16			

**Table 3:** Yield (% hemicelluloses) and sugar composition of hemicellulose fractions.

Samples	Yield ^1^	Ara ^2^	Gal ^2^	Glc ^2^	Xyl ^2^	GalA ^2^	GlcA ^2^	Ara ^2^/Xyl ^2^
WH-N ^3^	14.06	9.38	3.43	10.51	71.35	1.17	2.98	0.13
WH-U ^3^	16.96	10.36	3.51	10.11	69.05	1.19	2.76	0.15
AH-N ^3^	5.75	5.05	0.63	5.32	86.18	0.72	1.52	0.06
AH-U ^3^	6.09	5.47	0.84	4.34	85.83	0.72	1.46	0.06

^1^ % Hemicelluloses. ^2^ Abbreviations: Ara, arabinose; Gal, galactose; Glc, glucose; Xyl, xylose; GlcA, glucuronic acid; GalA, galacturonic acid. ^3^ Samples: WH-N, water-soluble hemicellulose, extracted under the conditions of KOH mass concentration of 3.7% and extraction temperature of 53 ℃; WH-U, water-soluble hemicellulose, extracted by ultrasound-assisted with ultrasonic treatment time of 28 min, KOH mass concentration of 3.7%, and extraction temperature of 53 ℃; AH-N, alkali-soluble hemicellulose, extracted under the conditions of KOH mass concentration of 3.7% and extraction temperature of 53 ℃; AH-U, alkali-soluble hemicellulose, extracted by ultrasound-assisted with ultrasonic treatment time of 28 min, KOH mass concentration of 3.7%, and extraction temperature of 53 ℃, so as follows.

**Table 4:** The content of residual lignin.

Samples	Residual Lignin Content (%)
WH-N	8.44%
WH-U	7.09%
AH-N	2.77%
AH-U	1.58%

**Table 5:** *M*_w_, *M*_n_, and polydispersity of hemicellulose.

Samples	*M*_w_^1^	*M*_n_^1^	Polydispersity Index ^1^
WH-N	31,585	22,484	1.40
WH-U	29,923	18,560	1.61
AH-N	75,991	56,531	1.34
AH-U	74,872	54,655	1.37

^1^*M*_w_: weight-average molecular weights of hemicellulose; *M*_n_: number-average molecular weights of hemicellulose; polydispersity index: weight-average molecular weights to number-average molecular weights.

## Appendix A

Table A1: Response surface analysis and level design, Table A2: Response surface design and experimental results.

**Table A1:** Response surface analysis and level design.

Samples	Factors		
	A/min	B/%	C/℃
−1	20	2	40
0	30	3	50
1	40	4	60

**Table A2:** Response surface design and experimental results.

No.	A/min	B/%	C/℃	Yield/%
1	20	2	50	15.88
2	40	2	50	14.51
3	20	4	50	19.70
4	40	4	50	18.69
5	20	3	40	16.33
6	40	3	40	17.20
7	20	3	60	20.53
8	40	3	60	17.56
9	30	2	40	16.57
10	30	4	40	20.31
11	30	2	60	17.02
12	30	4	60	22.65
13	30	3	50	22.39
14	30	3	50	22.22
15	30	3	50	21.61
16	30	3	50	22.10
17	30	3	50	21.38
